# Abnormal Blood Coagulation and Kidney Damage in Aged Hamsters Infected with Severe Acute Respiratory Syndrome Coronavirus 2

**DOI:** 10.3390/v13112137

**Published:** 2021-10-22

**Authors:** Marumi Ohno, Michihito Sasaki, Yasuko Orba, Toshiki Sekiya, Md. Abdul Masum, Osamu Ichii, Tatsuya Sawamura, Akemi Kakino, Yasuhiko Suzuki, Hiroshi Kida, Hirofumi Sawa, Masashi Shingai

**Affiliations:** 1 Laboratory for Biologics Development, International Institute for Zoonosis Control, Hokkaido University, Sapporo 001-0020, Japan; ohnom@czc.hokudai.ac.jp (M.O.); kida@vetmed.hokudai.ac.jp (H.K.); 2 Division of Molecular Pathobiology, International Institute for Zoonosis Control, Hokkaido University, Sapporo 001-0020, Japan; m-sasaki@czc.hokudai.ac.jp (M.S.); orbay@czc.hokudai.ac.jp (Y.O.); 3 International Collaboration Unit, International Institute for Zoonosis Control, Hokkaido University, Sapporo 001-0020, Japan; tsekiya@czc.hokudai.ac.jp; 4 Laboratory of Anatomy, Department of Basic Veterinary Sciences, Faculty of Veterinary Medicine, Hokkaido University, Sapporo 060-0818, Japan; vetmasum.sau@gmail.com (M.A.M.); ichi-o@vetmed.hokudai.ac.jp (O.I.); 5 Laboratory of Agrobiomedical Science, Faculty of Agriculture, Hokkaido University, Sapporo 060-0818, Japan; 6 Department of Molecular Pathophysiology, School of Medicine, Shinshu University, Matsumoto 390-8621, Japan; sawamura@shinshu-u.ac.jp (T.S.); kakinoa@shinshu-u.ac.jp (A.K.); 7 Division of Bioresources, International Institute for Zoonosis Control, Hokkaido University, Sapporo 001-0020, Japan; suzuki@czc.hokudai.ac.jp; 8 One Health Research Center, Hokkaido University, Sapporo 001-0020, Japan

**Keywords:** COVID-19, animal model, aged Syrian hamster

## Abstract

Systemic symptoms have often been observed in patients with coronavirus disease 2019 (COVID-19) in addition to pneumonia, however, the details are still unclear due to the lack of an appropriate animal model. In this study, we investigated and compared blood coagulation abnormalities and tissue damage between male Syrian hamsters of 9 (young) and over 36 (aged) weeks old after intranasal infection with severe acute respiratory syndrome coronavirus 2 (SARS-CoV-2). Despite similar levels of viral replication and inflammatory responses in the lungs of both age groups, aged but not young hamsters showed significant prolongation of prothrombin time and prominent acute kidney damage. Moreover, aged hamsters demonstrated increased intravascular coagulation time-dependently in the lungs, suggesting that consumption of coagulation factors causes prothrombin time prolongation. Furthermore, proximal urinary tract damage and mesangial matrix expansion were observed in the kidneys of the aged hamsters at early and later disease stages, respectively. Given that the severity and mortality of COVID-19 are higher in elderly human patients, the effect of aging on pathogenesis needs to be understood and should be considered for the selection of animal models. We, thus, propose that the aged hamster is a good small animal model for COVID-19 research.

## 1. Introduction

Since the end of 2019, coronavirus disease 2019 (COVID-19) has been a serious threat worldwide. Severe acute respiratory syndrome coronavirus 2 (SARS-CoV-2) is the causative pathogen for COVID-19. Given previous findings on sepsis-induced organ dysfunction [1], microvascular thrombosis is considered to contribute to tissue injury and multiple organ dysfunction syndrome also in COVID-19. In fact, hypercoagulable states demonstrated by increased D-dimer, elevated concentrations of fibrinogen and fibrinogen degradation products, and prolonged prothrombin time (PT) have been observed in patients with severe COVID-19, and the development of microthrombi in the small vessels of the lungs has been also reported [2,3,4,5]. In addition, systemic symptoms, such as kidney and liver dysfunction, have been reported to be associated with lethal outcomes in patients with COVID-19 [6]. However, the details of damage in extra-pulmonary organs are not fully understood, including whether it results from virus infection and replication in those tissues [7]. Therefore, the mechanisms of coagulation abnormalities and systemic symptoms in COVID-19 should be elucidated to better understand the pathogenesis of COVID-19. For this purpose, an appropriate animal model that reproduces these pathological events is needed but has yet been established. Although blood coagulation abnormalities have been reported in mice and dwarf hamsters after infection with SARS-CoV-2, animal models that exhibit organ damage other than the lungs have not been available [8,9,10]. Previous comparative studies in macaques and dwarf hamsters have demonstrated species, sex, and age differences in the pathogenesis of COVID-19 [10,11,12], suggesting the importance of considering experimental conditions of animals.

This present study investigates blood coagulation abnormalities and tissue damage caused by intranasal infection with SARS-CoV-2 in young (9-week-old) and aged (over 36-week-old) male Syrian hamsters since it was reported that male animals develop more severe COVID-19 than females [12]. Abnormal blood coagulation parameters and acute kidney damage were observed only in aged hamsters after the infection, although body weight loss and virus titers in the lungs were similar between the two age groups of animals. These findings indicate that age-related host factors may determine the severity of COVID-19 and emphasize the need for appropriate models, such as the use of aged animals, to understand disease pathogenesis.

## 2. Results

### 2.1. Prolongation of Prothrombin Time by SARS-CoV-2 Infection Only in Aged Hamsters

**Figure 1:**
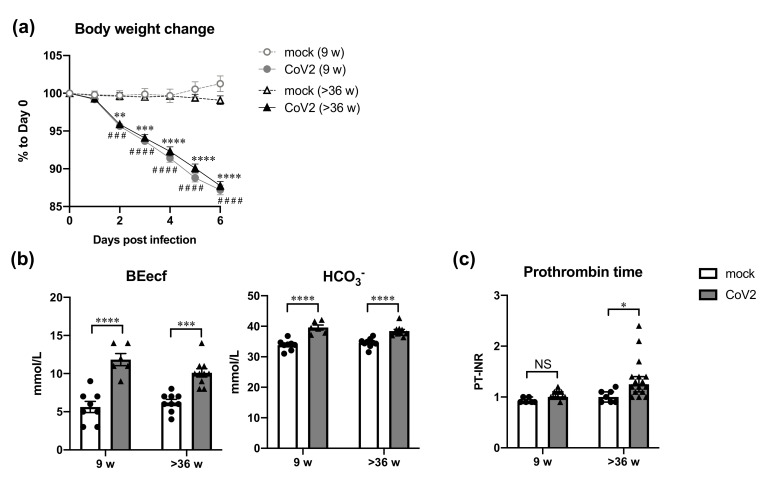
Changes of body weight and blood parameters in young and aged hamsters infected with SARS-CoV-2. Young (9 w) and aged (>36 w) hamsters were intranasally inoculated with PBS control (mock) or PBS containing SARS-CoV-2 virus (CoV2). Body weight was monitored daily until 6 days post infection (dpi) (**a**), and the hamsters were euthanized at 6 dpi for the collection of blood samples for measurement of acid-base status parameters BEecf and HCO^3−^ (**b**), and prothrombin time (**c**). (**a**) Symbols indicate the mean body weight ± SEM represented as a percentage of the original weight [mock (9 w), *n* = 7; CoV2 (9 w), *n* = 12; mock (>36 w), *n* = 7; CoV2 (>36 w), *n* = 17]. Circles and triangles indicate data from young and aged hamsters, respectively. Open and closed symbols indicate PBS control and virus-infected hamsters, respectively. ^###^ *p* < 0.001, ^####^ *p* < 0.0001 (young); ** *p* < 0.01, *** *p* < 0.001, **** *p* < 0.0001 (aged); two-way ANOVA with Tukey’s multiple comparison test. No significant difference was detected between young and aged hamsters at each time point. (**b**,**c**) White and gray bars indicate data from mock and CoV2-infected hamsters, respectively. Each dot indicates data from each individual. Bars indicate the mean ± SEM [(**b**) mock (9 w), *n* = 8; CoV2 (9 w), *n* = 6; mock (>36 w), *n* = 9; CoV2 (>36 w), *n* = 10] or the median ± interquartile; (**c**) 9 w-mock, *n* = 6; 9 w-CoV2, *n* = 12; >36 w-mock, *n* = 7; >36 w-CoV2, *n* = 16]. * *p* < 0.05, *** *p* < 0.001, **** *p* < 0.0001, two-way ANOVA with Sidak’s multiple comparison test. BEecf, base excess extracellular fluid; PT-INR, international normalized ratio of prothrombin time; NS, non-significant.

Intranasal infection with SARS-CoV-2 at a dose of 1.5 × 10^4^ plaque-forming units (PFU) caused significant body weight loss in young (9-week-old) and aged (over 36-week-old) hamsters at 2 days post-infection (dpi) and thereafter [*p* < 0.01, two-way analysis of variance (ANOVA)], as shown in Figure 1a. At 6 dpi, the average body weight was 87.2% ± 0.6% in young and 87.8% ± 0.6% in aged hamsters. No significant difference in body weight change was observed between young and aged hamsters at all the time points. At 6 dpi, the hamsters were sacrificed, and blood, serum, lung, liver, and kidney samples were collected to evaluate the effect of SARS-CoV-2 infection on the blood parameters and other tissues.

An imbalanced acid–base status was suggested in infected hamsters regardless of age at 6 dpi (Figure 1b). In both young and aged hamsters, base excess extracellular fluid (BEecf) and HCO_3_^−^ levels, known as metabolic alkalosis indicators, were elevated after virus infection (*p* < 0.0005, two-way ANOVA). With respect to virus infection-induced pneumonia, elevated BEecf and HCO_3_^−^ may have resulted from renal compensation for respiratory acidosis in the infected hamsters. Since coagulopathy has been reported in human patients with severe COVID-19 [2,3], the international normalized ratio of PT (PT-INR), a clinical index of the duration of blood coagulation, was evaluated. Interestingly, the median values of PT-INR of the aged hamsters were 1 in mock and 1.25 in infected animals, and a significant difference was observed despite the large individual variance (*p* < 0.05, two-way ANOVA; Figure 1c). In contrast, no clear difference existed in the PT-INR values of young hamsters between mock and infected animals (mock, 0.9; infected animals, 1.0; *p* = 0.6549, two-way ANOVA). Therefore, significant prolongation of PT by SARS-CoV-2 infection was a characteristic of the aged group, even though they showed the same level of weight loss as the young age group.

### 2.2. No Clear Effect of the Age on the Viral Replication and Lung Inflammation

**Figure 2:**
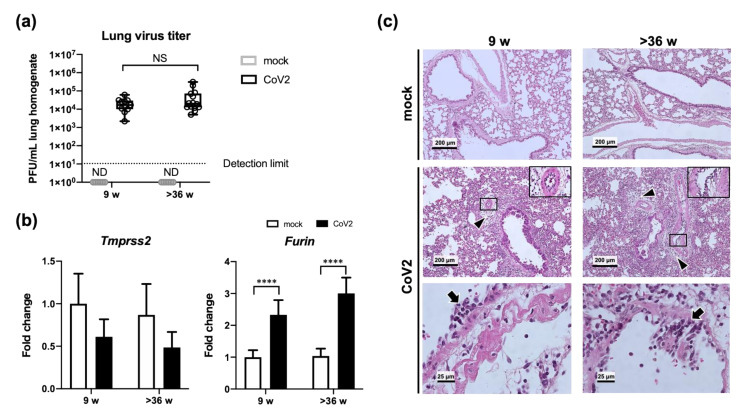
Virus titers and histopathological changes in the lungs of young and aged hamsters. Young (9 w) and aged (>36 w) hamsters were intranasally inoculated with PBS control (mock) or PBS containing SARS-CoV-2 virus (CoV2). Lung samples were collected at 6 days post-infection for the measurement of virus titers (**a**), gene expression analyses (**b**), and histopathological analyses (**c**). (**a**) Plaque assays on Vero-TMPRSS2 cells were performed to calculate the lung virus titers in each sample. Values are represented by box-and-whisker plots as follows: the central line in the box is the median, the bottom and top lines of the box are the first and third quartiles, respectively, whiskers are the minimum to maximum values. Gray and black boxes indicate data from mock and CoV2-infected hamsters, respectively. Each dot indicates data for each animal [mock (9 w), *n* = 6; CoV2 (9 w), *n* = 11; mock (>36 w), *n* = 6; CoV2 (>36 w), *n* = 14]. No significant difference was detected between young and aged hamsters in each treatment group by two-way ANOVA with Sidak’s multiple comparison test. NS, non-significant; ND, not detected. (**b**) Gene expression levels of *transmembrane protease, serine 2* (*Tmprss2*) and *Furin* are presented as the fold changes relative to those of young mock hamsters. Bars represent the mean ± SEM [mock (9 w), *n* = 6; CoV2 (9 w), *n* = 11; mock (>36 w), *n* = 6; CoV2 (>36 w), *n* = 14]. White and black bars indicate data from control and CoV2-infected hamsters, respectively. **** *p* < 0.0001, two-way ANOVA on dCt values with Sidak’s multiple comparison test. (**c**) For histopathological analyses, sections were stained with hematoxylin and eosin (HE). Arrowheads indicate leukocytes in the perivascular area. Inserts show swelling and detachment of vascular endothelial cells from the underlying basement membrane. Arrows indicate virus infection-induced vasculitis evident by leukocyte infiltration in the tunica intima and the tunica media.

Next, we examined whether or not viral load was affected by the age of the host. The plaque assay demonstrated a nearly equivalent number of infectious viruses in the lung homogenates of aged hamsters compared with that of young animals (two-way ANOVA; Figure 2a). The median values of lung virus titers at 6 dpi were 1.70 × 10^4^ PFU/mL (ranging from 0.22 × 10^4^ to 6.10 × 10^4^) in the young hamsters and 2.00 × 10^4^ PFU/mL (ranging from 0.50 × 10^4^ to 3.10 × 10^5^) in the aged animals. Quantitative real-time reverse transcription PCR (RT-PCR) was also performed to examine the age effect on expression levels of *Furin* and *transmembrane protease, serine 2* (*Tmprss2*), which have been reported to be essential for proteolytic activation of the SARS-CoV-2 spike protein in human airway cells [13]. A significant elevation of *Furin* expression was observed in the lungs of infected hamsters regardless of age (2.3-fold in young and 2.9-fold in aged hamsters, *p* < 0.0001, two-way ANOVA), although expression of *Tmprss2* was not significantly altered following virus infection (Figure 2b). Based on these results, the replication of SARS-CoV-2 in the lung is considered to be similar between young and aged male Syrian hamsters.

In addition, no apparent difference was found in the pathology of the lungs between young and aged hamsters (Figure 2c). The degree of lung inflammation caused by SARS-CoV-2 infection was examined in hematoxylin and eosin (HE)-stained sections from the hamsters sacrificed at 6 dpi. The lungs from both young and aged hamsters exhibited obvious peribronchial and perivascular inflammation, accumulation of inflammatory cells in the alveoli, thickened alveolar walls, and alveolar hemorrhage following virus infection (Figure 2c, middle panels, arrowheads). Swelling and detachment of vascular endothelial cells from the underlying basement membrane were also frequently observed in the lungs of infected hamsters (Figure 2c, middle panels, inserts). In addition, virus infection-induced vasculitis was evident as leukocyte infiltration into the tunica intima and the tunica media (Figure 2c, bottom panels, arrows). Furthermore, we evaluated the lung inflammation by scoring the size of the affected area according to a previous report [14], and no clear difference was found between the two age animal groups.

### 2.3. Comparison of Gene Expression Levels of Inflammatory- and Coagulation-Related Factors in the Lung, Liver, and Kidneys between Young and Aged Hamsters

**Figure 3:**
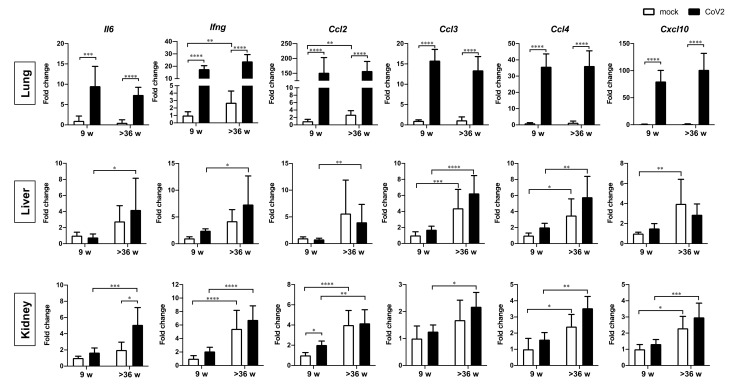
Gene expression of inflammatory factors in the lungs, liver, and kidneys of young and aged hamsters infected with CoV2. Young (9 w) and aged (>36 w) hamsters were intranasally inoculated with PBS control (mock) or PBS containing SARS-CoV-2 virus (CoV2), and the hamsters were euthanized at 6 days post infection for the collection of lung, liver, and kidney samples for measurement of the expression of inflammatory factor genes. Gene expression levels of *interleukin-6* (*Il6*), *interferon-gamma* (*Ifng*), *C-C motif chemokine 2* (*Ccl2*), *Ccl3*, *Ccl4*, and *C-X-C motif chemokine ligand 10* (*Cxcl10*) are presented as the fold changes relative to those of young mock hamsters. Bars represent the mean ± SEM [mock (9 w), *n* = 6; CoV2 (9 w), *n* = 11; mock (>36 w), *n* = 6; CoV2 (>36 w), *n* = 14]. White and black bars indicate data from control and CoV2-infected hamsters, respectively. * *p* < 0.05, ** *p* < 0.01, *** *p* < 0.001, **** *p* < 0.0001, two-way ANOVA on dCt values with Sidak’s multiple comparison test.

We also investigated the levels of gene expression related to inflammation and blood coagulation in the lung, liver, and kidney samples collected from mock and infected hamsters. The expression of *interleukin-6* (*Il6*), *interferon-gamma* (*Ifng*), *C-C motif chemokine 2* (*Ccl2*), *Ccl3*, *Ccl4*, and *C-X-C motif chemokine ligand 10* (*Cxcl10*) was measured by real-time RT-PCR (Figure 3). Elevated expression of inflammatory genes by SARS-CoV-2 infection was comparable in the lungs of young and aged hamsters. This is consistent with histological observations in the lung sections as described above. In contrast, the liver and kidney samples showed an increase in basal levels of most of these genes in aged hamsters, and the highest basal levels were found in the infected aged animals, although virus infection-dependent induction was not clear due to large individual variations. These results indicate that the liver and kidneys of aged hamsters are already in an inflammatory state, which is exacerbated by virus infection.

**Figure 4:**
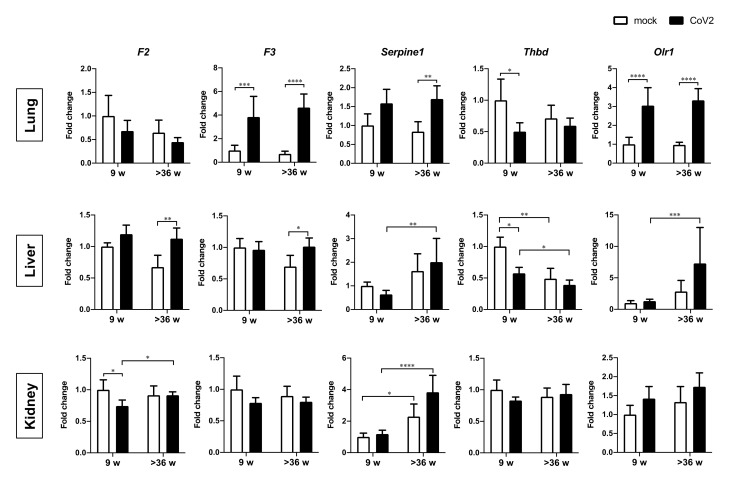
Gene expression of coagulation factors in the lungs, liver, and kidneys of young and aged hamsters infected with CoV2. Young (9 w) and aged (>36 w) hamsters were intranasally inoculated with PBS control (mock) or PBS containing SARS-CoV-2 virus (CoV2), and hamsters were euthanized at 6 dpi for the collection of lung, liver, and kidney samples for the measurement of coagulation-related factor gene expression. Gene expression levels of prothrombin-encoding *F2*, tissue factor-encoding *F3*, *serine protease inhibitor, clade E, member 1* (*Serpine1*), thrombomodulin-encoding *Thbd*, and *oxidized low-density lipoprotein receptor 1* (*Olr1*) are presented as the fold changes relative to those of young mock hamsters. Bars represent the mean ± SEM [mock (9 w), *n* = 6; CoV2 (9 w), *n* = 11; mock (>36 w), *n* = 6; CoV2 (>36 w), *n* = 14]. White and black bars indicate data from control and CoV2-infected hamsters, respectively. * *p* < 0.05, ** *p* < 0.01, *** *p* < 0.001, **** *p* < 0.0001, two-way ANOVA on dCt values with Sidak’s multiple comparison test.

In addition to inflammatory cytokines/chemokines, the expression levels of coagulation-related factors, prothrombin-encoding *F2*, tissue factor-encoding *F3*, and plasminogen activator inhibitor type 1 (PAI-1) encoding *serine protease inhibitor, clade E, member 1* (*Serpine1*) were upregulated by virus infection in the lungs and/or liver of aged hamsters (Figure 4). The expression of the *F3* gene in the lungs was also increased by infection in the young hamsters to a similar level as the aged animals. The expression of anticoagulant factor thrombomodulin-encoding *Thbd* showed a tendency to be reduced by virus infection in the lungs and liver of young hamsters, whereas the basal expression of this gene in the livers of aged hamsters was low prior to infection. We also evaluated the gene expression of *oxidized low-density lipoprotein receptor 1* (*Olr1*), which encodes lectin-like oxidized low-density lipoprotein receptor-1 (LOX-1) that is involved in prothrombotic change of the vascular wall by promoting the attachment of activated platelets to endothelial cells, endothelial dysfunction, and severe influenza-associated thrombus formation in the lung [15,16,17]. The expression of *Olr1* was increased in the lung by virus infection, with similar induction levels in young and aged hamsters. In the liver, however, the *Olr1* expression after the infection was significantly higher in aged animals than that in young ones. The overall effect of SARS-CoV-2 infection was considered to be a change in the direction of accelerated thrombogenesis, particularly in the liver of aged hamsters, which is the most important source of coagulation-related factors.

### 2.4. SARS-CoV-2 Infection-Associated Acute Kidney Damage in Aged Hamsters

**Figure 5:**
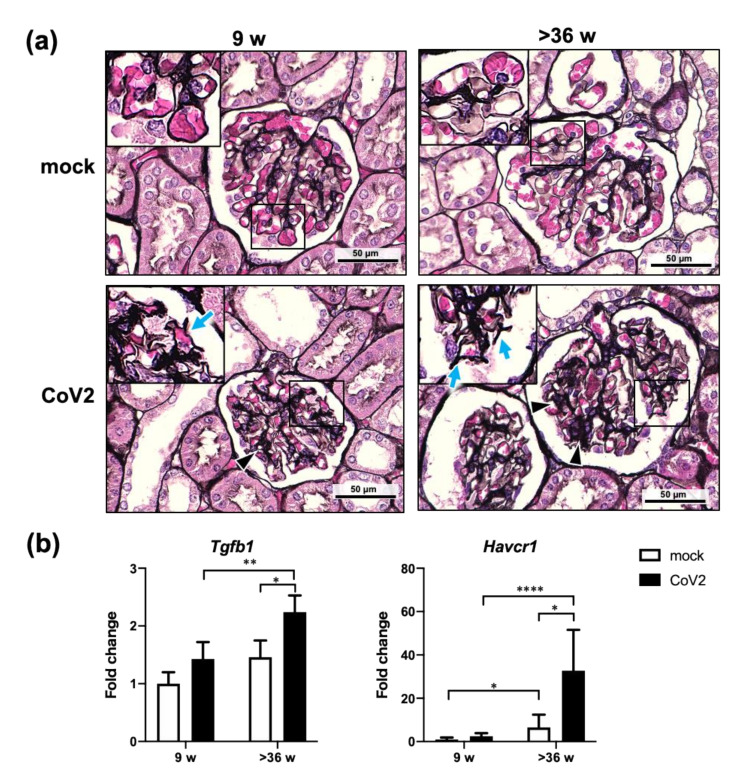
Pathological changes in the kidneys from young and aged hamsters infected with CoV2. Young (9 w) and aged (>36 w) hamsters were intranasally inoculated with PBS control (mock) or PBS containing SARS-CoV-2 virus (CoV2), and hamsters were euthanized at 6 days post infection for the collection of kidney samples for histopathological analyses (**a**) and gene expression analyses (**b**). (**a**) For histopathological analyses, kidney sections were stained with periodic acid–methenamine silver (PAM). Arrowheads indicate increased mesangial substrate. Arrows in the insert panels indicate wrinkle changes in the glomerular basement membranes. (**b**) Gene expression levels of *transforming growth factor beta 1* (*Tgfb1*) and *hepatitis A virus cellular receptor 1* (*Havcr1*) are presented as the fold changes relative to those of young mock hamsters. Bars represent the mean ± SEM [mock (9 w), *n* = 6; CoV2 (9 w), *n* = 10; mock (>36 w), *n* = 6; CoV2 (>36 w), *n* = 13]. White and black bars indicate data from mock and CoV2-infected hamsters, respectively. * *p* < 0.05, ** *p* < 0.01, **** *p* < 0.0001, two-way ANOVA on dCt values with Sidak’s multiple comparison test.

Since the gene expression analysis suggested inflammation in the kidneys in the aged-infected group, renal histological analysis was performed (Figure 5). In the infected young and aged hamsters, periodic acid-methenamine silver (PAM)-stained kidney sections revealed changes that indicate glomerular endothelial cell injury, such as wrinkles in the glomerular basement membrane (Figure 5a, arrows in inserts) and increased mesangial matrices (Figure 5a, arrowheads). These pathological changes were more pronounced in the aged hamster group. In addition, renal expression of kidney injury markers was assessed by real-time RT-PCR (Figure 5b). Increased mesangial matrices in aged-infected hamsters were further suggested by increased expression of the *transforming growth factor beta 1* (*Tgfb1*) gene [18]. SARS-CoV-2 infection also resulted in increased expression of *hepatitis A virus cellular receptor 1* (*Havcr1*), a sensitive and specific marker for renal proximal tubule damage, in the kidneys of aged hamsters [19]. Overall, the results suggest that SARS-CoV-2 infection induces significant renal damage particularly in aged hamsters.

### 2.5. Increased Intravascular Fibrin Deposition in the Lungs of Aged Hamsters after SARS-CoV-2 Infection

**Figure 6:**
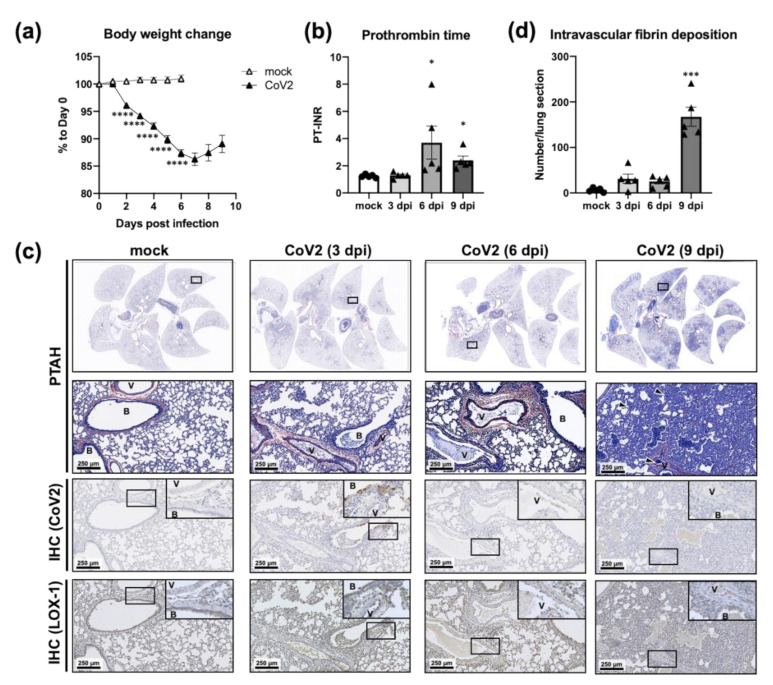
Increased intravascular fibrin deposits in the lungs of aged hamsters infected with CoV2. Aged (>36 w) hamsters were intranasally inoculated with PBS control (mock) or PBS containing SARS-CoV-2 virus (CoV2). Body weight was monitored daily until sacrifice (**a**), and blood and lung samples were collected at 3, 6, and 9 days post infection (dpi) for the measurement of prothrombin time (**b**), histopathological analyses (**c**), or measurement of the number intravascular fibrin deposits (**d**). (**a**) Open and closed triangles indicate the mean body weight ± SEM represented as a percentage of the original weight of PBS control and virus-infected hamsters (combined data from animals sacrificed at 3, 6, and 9 dpi), respectively (*n* = 5). **** *p* < 0.0001; multiple t-test using the Holm-Sidak’s method for a multiple comparison correction. (**b**,**d**) Bars represent the mean ± SEM (*n* = 5). White and gray bars indicate data from mock and CoV2-infected hamsters, respectively. * *p* < 0.05, *** *p* < 0.0001, one-way ANOVA with Dunnett’s multiple comparison test (vs. mock hamsters). PT-INR, international normalized ratio of prothrombin time. (**c**) For histopathological analyses, lung sections were stained with phosphotungstic acid hematoxylin (PTAH). Arrowheads in the PTAH-stained section of CoV-2 infected lung (9 dpi) indicate intravascular fibrin deposits. Immunohistochemistry (IHC) was further performed to detect N protein of SARS-CoV-2 and LOX-1. V, vessel; B, bronchus.

Since the above results suggest that the SARS-CoV-2 infection causes more severe disease in aged hamsters, we further analyzed the effect of the infection with SARS-CoV-2 at a dose of 5.0 × 10^4^ PFU on the blood coagulation system and the expression of inflammatory- and coagulation-related genes utilizing samples collected from aged hamsters at 3, 6, and 9 dpi. Significant body weight loss was observed in aged hamsters at 2 dpi and thereafter (*p* < 0.00001, multiple *t*-test) with a recovery trend after 6–7 dpi (Figure 6a). The mean values of PT-INR were 1.24 ± 0.05 (mock), 1.30 ± 0.10 (3 dpi), 3.70 ± 1.22 (6 dpi), and 2.40 ± 0.31 (9 dpi) as shown in Figure 6b. Significant prolongation of PT was observed at 6 and 9 dpi (*p* < 0.05, one-way ANOVA).

To investigate the thrombus formation in the lung after infection with SARS-CoV-2, histological analyses were conducted. In serial lung sections, intravascular fibrin deposits were visualized by phosphotungstic acid hematoxylin (PTAH) staining (upper panels, Figure 6c), and N protein of SARS-CoV-2 and LOX-1 were detected by immunohistochemistry (IHC; lower panels, Figure 6c). Intravascular fibrin deposits were commonly observed in the inflamed areas of infected animals, and the number on the sections showed an increasing trend at 3 and 6 dpi and a significant increase at 9 dpi (the mean values: 6.40 ± 1.91, mock; 31.20 ± 10.26, 3 dpi; 25.00 ± 5.36, 6 dpi; 167.40 ± 21.04, 9 dpi; one-way ANOVA; Figure 6d). At 3 dpi, mild thickening of the alveolar walls around the bronchi was observed, and positive reactions for the viral antigen were detected in the epithelium of bronchi and alveoli. At 6 dpi, a more advanced inflammatory response was observed, but the viral antigen was only found sporadically in the alveolar epithelium. On day 9 after infection, the hamsters had developed lesions with focal and/or multiple focal areas of inflammation in all lobes although there was no detectable reaction to the antigen. Furthermore, no viral antigen was detected in the perivascular tissues and endothelium in the lungs of infected hamsters at all time points. On the other hand, LOX-1 was mainly expressed in epithelial cells before infection, but after virus infection, it was strongly expressed in vascular endothelial cells and vascular smooth muscle in addition to these cells. The increased expression of LOX-1 in vascular endothelium and proliferated alveolar epithelial cells was confirmed even on days 6 and 9 when the viral antigen was hardly detected in the lungs. These results indicate that the infection with SARS-CoV-2 is not a direct cause of vasculitis or thrombus formation in the lungs. Rather, host factor(s), such as induced LOX-1 in the vascular endothelium, may be associated with it as a second messenger.

### 2.6. Gene Expression of Inflammatory Factors in the Aorta, Liver, and Kidneys of Aged Hamsters after SARS-CoV-2 Infection

**Figure 7:**
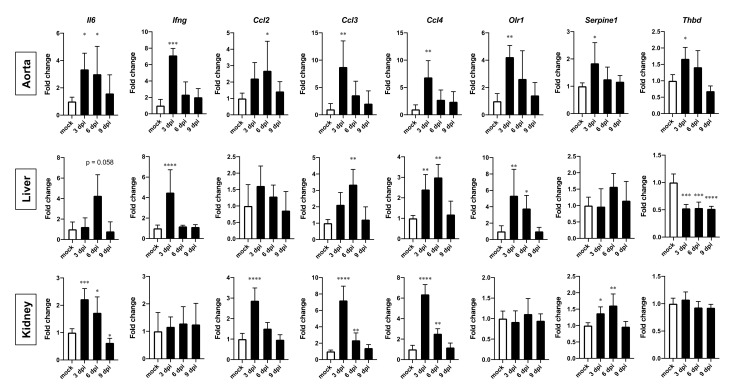
Gene expression of inflammation and coagulation factors in the aorta, liver, and kidneys of aged hamsters infected with CoV2. Aged (>36 w) hamsters were intranasally inoculated with PBS control (mock) or PBS containing SARS-CoV-2 virus (CoV2), and hamsters were euthanized at 3, 6, and 9 days post infection (dpi) for the collection of aorta, liver, and kidney samples for gene expression analyses. Gene expression levels of *interleukin-6* (*Il6*), *interferon-gamma* (*Ifng*), *C-C motif chemokine 2* (*Ccl2*), *Ccl3*, *Ccl4, oxidized low-density lipoprotein receptor 1* (*Olr1*), *serine protease inhibitor, clade E, member 1* (*Serpine1*), and thrombomodulin-encoding *Thbd* are presented as the fold changes relative to those of mock hamsters. Bars represent the mean ± SEM (*n* = 5). White and black bars indicate data from control and CoV2-infected hamsters, respectively. * *p* < 0.05, ** *p* < 0.01, *** *p* < 0.001, **** *p* < 0.0001, one-way ANOVA on dCt values with Dunnett’s multiple comparison test (vs. mock hamsters).

Quantitative real-time RT-PCR was also performed to measure the levels of inflammatory- and coagulation-related factors in the aorta, liver, and kidneys (Figure 7). Gene expression of inflammatory factors (*Il6*, *Ifng*, *Ccl2*, *Ccl3*, and *Ccl4*) was clearly increased in the aorta, liver, and kidneys at 3 and/or 6 dpi. In addition, the aorta and liver demonstrated a significant increase in *Olr1* gene expression at 3 dpi. The induction of *Serpine1* was observed in the aorta and kidneys at 3 and/or 6 dpi. On the other hand, the liver showed a significant decrease in *Thbd* at 3, 6, and 9 dpi, suggesting a continuous decrease in thrombomodulin production in the liver after the infection.

### 2.7. Proximal Urinary Tract Damage and Mesangial Matrix Expansion in the Kidneys of Aged Hamsters after SARS-CoV-2 Infection

**Figure 8:**
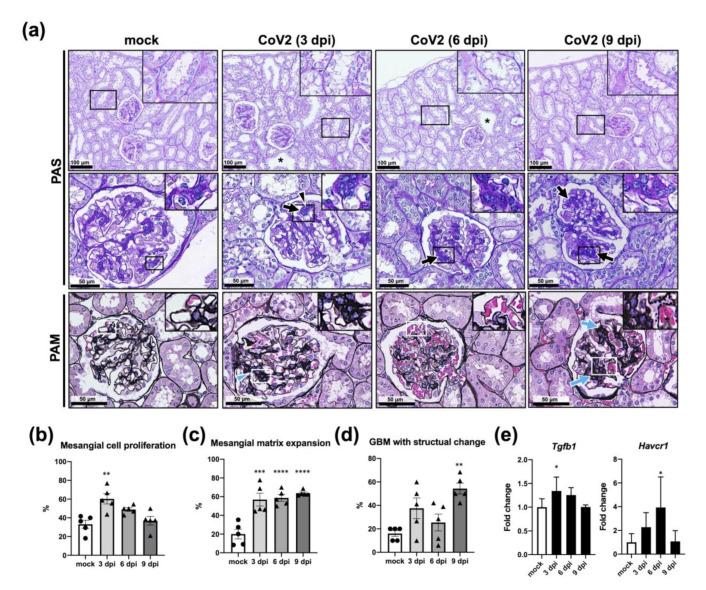
Pathological changes in the kidneys from aged hamsters infected with CoV2 at 3, 6, and 9 days post infection (dpi). Aged (>36 w) hamsters were intranasally inoculated with PBS control (mock) or PBS containing SARS-CoV-2 virus (CoV2), and hamsters were euthanized at 3, 6, or 9 dpi for the collection of kidney samples for histopathological analyses (**a**–**d**) and gene expression analyses (**e**). (**a**) For histopathological analyses, kidney sections were stained with periodic acid-Schiff (PAS) and periodic acid–methenamine silver (PAM). Asterisks indicate dilated urinary tracts. Arrowheads indicate proliferated mesangial cells. Arrows indicate increased mesangial matrix. (**b**–**d**) In each section, 10–20 glomeruli that are close to the largest diameter where the vascular and ureteric poles were seen were selected. The percentage of glomeruli with mesangial cell proliferation (**b**), mesangial matrix expansion (**c**), and glomerular basement membrane (GBM) showing morphological changes (**d**) was calculated. Bars represent the mean ± SEM (*n* = 5). ** *p* < 0.01, *** *p* < 0.001, **** *p* < 0.0001, one-way ANOVA with Dunnett’s multiple comparison test (vs. mock hamsters). (**e**) Gene expression levels of *transforming growth factor beta 1* (*Tgfb1*) and *hepatitis A virus cellular receptor 1* (*Havcr1*) are presented as the fold changes relative to those of mock hamsters. Bars represent the mean ± SEM (*n* = 5). White and black bars indicate data from mock and CoV2-infected hamsters, respectively. * *p* < 0.05, one-way ANOVA on dCt values with Dunnett’s multiple comparison test (vs. mock hamsters).

Since gene expression of inflammatory factors was high in the early stages of infection in the kidneys, renal histological analyses were performed. In Figure 8a, periodic acid-Schiff (PAS)-stained sections demonstrated that loss of the proximal tubular brush border was observed at 3 and 6 dpi, but not at 9 dpi. As for histological changes in the glomerulus, proliferation of mesangial cells was observed at 3 dpi, and expansion of the mesangial matrices was observed in all infected groups. The frequency of those observations was evaluated by counting numbers in the glomeruli where both vascular and tubular poles were visible in PAS-stained sections, and the percentage was calculated for each group. The percentage of glomeruli with proliferated mesangial cells was 33.13 ± 4.29 (mock), 60.34 ± 5.55 (3 dpi), 48.89 ± 1.93 (6 dpi), and 36.90 ± 4.62 (9 dpi), and a significant difference was detected in the value of 3 dpi group in comparison with mock (*p* < 0.01, one-way ANOVA; Figure 8b). The percentage of glomeruli with expanded mesangial matrices was 20.00 ± 5.21 (mock), 56.84 ± 6.87 (3 dpi), 58.74 ± 3.63 (6 dpi), and 63.69 ± 1.29 (9 dpi), and a significant difference was detected in the values of 3, 6, and 9 dpi groups in comparison with mock (*p* < 0.005, one-way ANOVA; Figure 8c). Morphological changes in the glomerular basement membrane were prominent at 9 dpi (PAM-stained sections, Figure 8a). The percentage of glomeruli showing morphological change in basement membrane was 16.00 ± 2.45 (mock), 37.57 ± 8.80 (3 dpi), 25.44 ± 7.20 (6 dpi), and 54.33 ± 4.65 (9 dpi), and a significant difference was detected in the value of 9 dpi group in comparison with mock (*p* < 0.01, one-way ANOVA; Figure 8d). These observations, proximal tubular damage, and mesangial matrix expansion were further supported by increased expression of *Havcr1* at 6 dpi and *Tgfb1* at 3 dpi, respectively (Figure 8e).

Despite these histological changes and increased gene expression of inflammatory factors in the kidneys, no viral antigens were detected by immunohistochemistry in any of the infected animals (data not shown). Thus, the pathological changes observed in the kidneys of SARS-CoV-2 infected hamsters are not directly attributable to the virus infection as previously reported [20] but are probably due to systemic biological reactions at least under our experimental conditions. Aged hamsters could be an animal model to represent kidney damage which is frequently observed in COVID-19 patients [21].

## 3. Discussion

In this study, blood coagulation abnormalities and tissue damage caused by SARS-CoV-2 infection were compared between young and aged male Syrian hamsters. The virus titers, inflammation, and changes in gene expression of inflammatory factors in the lungs at 6 dpi were comparable in the hamsters of both age groups. These results were consistent with those in previous studies that demonstrated no clear difference in the virus titers in respiratory tissues between 4–6-week-old and 28–34-week-old Syrian hamsters at 3–14 dpi [14,22]. In addition, in the previous study, no significant effect of aging on inflammation and immune response was evident in the lungs within 5 days of the infection with SARS-CoV-2, although they appear more pronounced as days pass after infection (e.g., 14 dpi) [22]. It has also been reported that histopathological changes caused by SARS-CoV-2 infection in the lungs of rhesus macaques older than 15 years at 6 dpi were similar to those of 3-year-old macaques [11]. Therefore, the effect of aging on the inflammatory responses in the lungs may be minor during the early stage of infection, although acquired immune responses, such as the induction of neutralizing antibodies, may have been influenced by the age as reported in monkey models [11].

Although there was no apparent effect of age on body weight loss after infection as well as virus titers and inflammatory responses in the lungs, prolongation of PT was observed only in the aged hamsters in this study. In the subsequent experiment that examined blood coagulation abnormalities at different time points in aged animals, intravascular blood coagulation, which was indicated by increased fibrin deposition in the lungs, tended to increase from 3 dpi and was frequently observed at 9 dpi. On the other hand, PT prolongation was observed at 6 and 9 dpi, suggesting that the virus infection caused increased blood coagulation, followed by consumption of coagulation factors. In addition, the decreases in *Thbd* gene expression observed in the liver at 3, 6, and 9 dpi may have resulted in a shortage of anticoagulant thrombomodulin and facilitated a thrombosis tendency. Considering that thrombosis occurs even in asymptomatic COVID-19 patients [23], it is presumed that the virus infection activates a thrombogenic pathway and continued and/or severe thrombosis leads to the consumption of coagulation factors, resulting in prolongation of PT. This notion is strongly supported by a recent report demonstrating positive correlations of Sequential Organ Failure Assessment score with the delay of thrombin generation, plasmin generation, and fibrinolysis in COVID-19 patients, indicating a consumptive coagulopathy at later stages of severe COVID-19 [24]. Therefore, the aged hamster model should be useful to understand the pathogenesis of abnormal blood coagulation in human COVID-19 patients.

Since intravascular fibrin deposits were observed mainly in inflamed areas of the lungs, inflammation is considered to be involved in the promotion of abnormal blood clotting. Although infected hamsters showed the most pronounced lung lesions at 9 dpi, no viral antigen was detected even in the epithelium of the bronchi and alveoli at that time point. Furthermore, the absence of viral antigens in the vascular endothelium at any time point suggests that virus infection or replication is not the direct cause of vasculitis or intravascular blood clotting in the lungs of infected hamsters. This notion is supported by a previous report showing the involvement of not the virus itself but neutrophil extracellular traps (NETs) in vasculitis in the lung using the hamster COVID-19 model [25]. Recently, our group reported a critical role of LOX-1 in thrombin generation and lung thrombosis in a severe influenza mouse model [17]. As previously observed in mice infected with the influenza virus, the induction of the *Olr1* gene was also detected in the lungs, liver, and aorta of hamsters after SARS-CoV-2 infection in this study. In addition, immunohistochemistry demonstrated that LOX-1 increased in the bronchial epithelial and vascular endothelium of the lungs after infection with SARS-CoV-2. Given that the influenza-associated lung thrombosis did not occur in *Olr1* knockout mice [17], it is possible that LOX-1 is also involved in promoting the thrombus formation in COVID-19.

In addition to prolonged PT, more pronounced kidney damage after infection with SARS-CoV-2 was a characteristic feature of aged hamsters. As previously reported [20], viral antigens were not detected in the kidneys in this study, so the pathological changes in the kidneys should not be directly caused by virus infection or replication. In the experiment that followed histological changes in the kidneys over time, proximal urinary tract damage was observed at 3 and 6 dpi, and the significant increase in *Havcr1* gene at 6 dpi may indicate that repair of damaged proximal urinary tract occurred. In addition, the proliferation of mesangial cells was observed only in the early stage of infection, while expansion of the mesangial matrix was found in all infected hamsters from 3 to 9 dpi. Given that extracellular matrix deposition in the glomeruli is promoted by PAI-1 [26], the prolonged mesangial matrix expansion could be associated with the increased gene expression of *Serpine1*, which encodes PAI-1, in the kidney of aged hamsters infected with SARS-CoV-2 as well as the induction of *Tgfb1*. Since prolonged mesangial matrix expansion leads to the development of glomerulosclerosis [27], aged hamsters could be used as a model for the study of COVID-19-associated glomerulosclerosis [6].

So far, hamsters, mice, monkeys, and ferrets, have been used for research on COVID-19, but no model has been developed to reproduce renal damage, such as acute tubular injury and glomerulosclerosis, that frequently occurs in COVID-19 patients [6]. Mice that express human angiotensin-converting enzyme 2 (hACE2), a SARS-CoV-2 receptor of the host, have been thought to be a good animal model for COVID-19 with elevated PT and D-dimer as well as cytokine induction following SARS-CoV-2 infection [8]. However, even the 6–11-month-old mice did not show apparent kidney abnormalities after SARS-CoV-2 infection, although highly expressed hACE2 and viral genome were detected in the tissue [8,9]. Therefore, species differences should not only be considered based on viral receptors and cytokine response but also physiological features to explore the disease mechanisms. For example, hamsters, like humans, have a chymase pathway for the synthesis of angiotensin II. Considering the potential role of angiotensin II in the pathogenesis of COVID-19 [28] and age-dependent increase of the activity [29], the use of hamsters, especially aged animals for COVID-19 research seems a rational approach, because their metabolic pathway for angiotensin II is similar to that of humans, at least among rodents.

In summary, we compared the pathogenesis of COVID-19 between young and aged male Syrian hamsters. Aged hamsters, but not young animals, demonstrated prolongation of PT, intravascular clotting, and acute kidney damage upon SARS-CoV-2 infection, which are often observed in patients with COVID-19 and well associated with disease severity and mortality [21]. Thus, we propose that the aged hamster is a good small animal model for COVID-19 research.

## 4. Materials and Methods

### 4.1. Materials

PBS, Dulbecco’s Modified Eagle’s Medium (DMEM), and fetal bovine serum (FBS) were purchased from Gibco/Life Technologies (Carlsbad, CA, USA). 

### 4.2. Virus and Cell

TMPRSS2-expressing Vero E6 cells (Vero-TMPRSS2) were established as previously reported [30]. SARS-CoV-2 (JPN/TY/WK-521, EPI_ISL_408667) isolated from a COVID-19 patient was kindly provided by the National Institute of Infectious Diseases in Tokyo, Japan. Vero-TMPRSS2 were infected with SARS-CoV-2 in DMEM supplemented with 2% FBS and incubated at 37 °C for 48 h. Cell culture media was collected and centrifuged at 500× *g* for 5 min to obtain virus-containing supernatant. Aliquots of the collected supernatant were stored at −80 °C. Plaque assays using the same cell line were performed to determine the virus titer.

### 4.3. Hamster

Young (9 weeks old) and aged (over 36 weeks old, retired from bleeding) male Syrian hamsters were purchased from Japan SLC (Shizuoka, Japan) and housed in a BSL-3 laboratory at the International Institute for Zoonosis Control, Hokkaido University, under standard laboratory conditions (room temperature 22 °C ± 2 °C, relative humidity 50% ± 10%) and a 12/12 h light/dark cycle. The hamsters were administered a standard CE-2 chow diet purchased from CLEA Japan (Sapporo, Japan) with water ad libitum. Experiments were performed on 9- and over 36-week-old hamsters.

### 4.4. Virus Infection and Sample Collection

SARS-CoV-2 virus particles at 1.5 × 10^4^ PFU (comparative study between young and aged hamsters) or 5 × 10^4^ PFU (time-course study using aged hamsters) in 200 µL of PBS or PBS only (mock) were intranasally inoculated into the hamsters under inhalation anesthesia with isoflurane. Body weight loss was monitored daily after infection, and 25% weight loss was defined as a humane endpoint. At time points indicated in the text, the hamsters were euthanized with an overdose of isoflurane followed by cervical dislocation, and their lungs, liver, kidneys, and blood samples were collected. Blood samples were used for the measurement of coagulation parameters and blood chemistry tests. Tissue samples from some hamsters were transferred into tubes containing Maskedform (Japan Tanner, Osaka, Japan) for histopathological analysis and fixed at room temperature for 7–10 days. Small pieces of tissue samples from other hamsters were transferred into tubes containing 750 μL of TRIzol (Thermo Fisher Scientific, Waltham, MA, USA), zirconium, and stainless beads and homogenized with a Micro Smash MS-100R (TOMY, Tokyo Japan) at 4500 rpm for 60 s. Homogenized tissue samples in TRIzol were stored at −20 °C until RNA extraction. In a comparative study between young and aged hamsters, lung samples were homogenized in PBS using a TissueRupter (QIAGEN, Valencia, CA, USA), and 50 μL of the homogenate was transferred to a tube containing 450 μL TRIzol LS and stored at −20 °C until RNA extraction. The remaining lung homogenates were centrifuged at 2200× *g* for 5 min, and the supernatants were collected and stored at −80 °C for plaque assays. In a time-course study, whole lungs collected from each animal were used for histopathological analyses.

### 4.5. Assessment of Blood Acid–Base Status

Whole-blood samples collected from the hamsters were immediately used for blood acid–base assessment with an i-STAT 1 analyzer (Abbott Laboratory, North Chicago, IL, USA) using a CG4+ cartridge (03P85-25, Abbott Laboratories). Briefly, venous blood (2–4 mL) was collected from the vena cava of sacrificed hamsters using a 5 mL syringe, and 105 μL of whole blood was loaded into the cartridge. After sealing, the cartridge was inserted into the i-STAT 1 analyzer for measurement.

### 4.6. Measurement of Coagulation Parameters

Whole-blood samples collected from the hamsters were immediately used for PT-INR measurements using a CoaguChek Pro II (Roche Diagnostics, Mannheim, Germany) and a PT test strip. 

### 4.7. Measurement of Gene Expression Using Real-Time RT-PCR

Total RNA was extracted from homogenized tissue samples in TRIzol using the Direct-zol RNA Miniprep Kit (ZYMO Research, Orange, CA, USA) followed by cDNA synthesis using High-Capacity cDNA Reverse Transcription Kits (Thermo Fisher Scientific) according to the manufacturer’s instructions. The expression of *Furin*, *Tmprss2*, *Il6*, *Ifng*, *Ccl2*, *Ccl3*, *Ccl4*, *Cxcl10*, *Serpine1*, *F2*, *F3*, *Thbd*, *Olr1,* *Havcr1,* and *Tgfb1* was quantified using real-time PCR with a StepOne Real-Time PCR System (Applied Biosystems, Foster City, CA, USA) using custom-made TaqMan probes (Applied Biosystems), and the relative expression was calculated using the comparative Ct method. The housekeeping genes used for the normalization were selected for each organ from *TATA-box binding protein* (*Tbp*), *β-Actin* (*Actb*), *cyclin-dependent kinase inhibitor 1B* (*Cdkn1b1*), and *18S* that showed small differences among the groups and Ct values close to those of the target genes (*Cdkn1b* for lung and *Actb* for other tissues). Supplemental Table S1 provides the sequences of the primers and TaqMan probes.

### 4.8. Plaque Assay

Plaque assay was performed for the evaluation of lung virus titers as previously reported [30].

### 4.9. Histopathological Analysis

After the hamsters were euthanized, their lung and kidney samples were collected, immersion fixed in Maskedform (Japan Tanner, Osaka, Japan) for 7–10 days at room temperature, dehydrated, embedded in paraffin using a Tissue-Tek VIP (Sakura, Tokyo, Japan), and cut into 3 μm slices. Lung sections were stained with HE or PTAH after dewaxing in xylene and rehydrating in decreasing ethanol concentrations. Kidney sections were stained with PAM or PAS and the effect of the infection on the glomerular condition was evaluated as follows. If the maximum number of mesangial cells in a mesangial region was greater than 4, the glomerulus was considered to have mesangial cell proliferation. Each mesangial matrix area was checked on PAS-stained sections. If a glomerulus had two or more mesangial regions that were at least as wide as two mesangial cell nuclei, the glomerulus was considered to have an expanding mesangial substrate. The glomerular basement membrane was checked on PAM-stained sections. If the spike-like extensions of the basement membrane were observed, the glomerulus was considered to have structural changes in the basement membrane.

### 4.10. Anti-LOX-1 Antibody Preparation

The antibody against hamster LOX-1 was selected from 6 anti-LOX-1 monoclonal antibodies previously developed by immunizing other species’ LOX-1; TS92, TS20, TS58, HUC52, HUC3-48, and HUC5-40 [31,32,33,34]. Briefly, Golden hamster *Olr1* cDNA (accession no. XM_021223625.1) was synthesized by Eurofins Genomics (Tokyo, Japan) and subcloned into pEF6/V5/His vector (Thermo Fisher Scientific). The expression vector was transfected into COS-7 cells on a 384-well plate using Lipofectamin 2000 (Invitrogen, Waltham, MA, USA). After 48 h, cells were immunostained with 6 clones of the anti-LOX-1 antibodies in conjunction with Alexa488-labeled anti-IgG second antibody (1:2000, Thermo Fisher Scientific) to examine cross-reactivity to hamster LOX-1. HUC52 and TS20 were found to cross-react with hamster LOX-1, and HUC52, which showed higher reactivity, was selected for the present study.

Furthermore, based on the amino acid sequence of the light and heavy chain variable regions of the HUC52, codon-optimized cDNAs for Chinese Hamster Ovary (CHO) cells were designed and synthesized by GenScript Corp. (Piscataway, NJ, USA). The sequence was introduced into an expression vector pDC62c5-U533 (PCT/JP2019/18899) which possesses the humanized signal peptide cDNAs, and the expression vector was transfected into CHO cell line DG44 by utilizing FreeStyle™ MAX Reagent (Thermo Fisher Scientific) to obtain stable cell lines. Two clones highly expressing HUC52 were selected, and the culture conditions using a protein-free medium were optimized to produce 2 g/L of HUC52 for 14 days. The highly purified HUC52 was prepared from culture supernatant using a protein A affinity column chromatography and ion-exchange column chromatography.

### 4.11. Immunohistochemistry

Immunohistochemistry was performed on unstained lung sections to detect viral antigens and LOX-1. After deparaffinization, antigen retrieval was performed with 20 mM Tris buffer (pH 9.0) for 15 min at 110 °C. The sections were soaked in methanol containing 0.3% H_2_O_2_ for 20 min at room temperature to block internal peroxidase activity. After blocking in 10% goat serum for 1 h at room temperature, samples were probed with anti-Nucleocapsid protein of SARS-CoV-2 (1:1000, HL344, rabbit monoclonal antibody; GeneTex, Irvine, CA, USA) or anti-LOX-1 antibody (1:2000, HUC52) at 4 °C overnight. After washing in PBS three times, the sections were incubated with biotin-conjugated goat anti-rabbit IgG antibody [1:100, SABPO(R) Kit, Nichirei, Tokyo, Japan] for the viral antigen or anti-human IgG (1:200, ab97223, Abcam) for LOX-1 for 30 min at room temperature. After washing in PBS three times, the sections were incubated with streptavidin-biotin complex [SABPO(R) Kit, Nichirei] for 30 min, followed by with diaminobenzidine solution. Counterstaining was performed with hematoxylin solution.

### 4.12. Statistical Analysis

Statistical analyses were performed using Prism 8 (GraphPad Software, San Diego, CA). Differences were determined using a two-way ANOVA or one-way ANOVA with appropriate multiple comparison tests and were considered significant at *p* < 0.05. The Anderson–Darling test was used to determine the normality. Data that are normally distributed are presented as the mean ± standard error of the mean (SEM), and those that are not are presented as the median ± quartiles.

## Data Availability

The data presented in this study are available on request from the corresponding author.

## Supplementary Materials

Supplemental Table S1 is available online at https://www.mdpi.com/article/10.3390/v13112137/s1. Table S1. Sequences of the primers and TaqMan probes.
